# Autophagy processes are dependent on EGF receptor signaling

**DOI:** 10.18632/oncotarget.25708

**Published:** 2018-07-13

**Authors:** Vincenzo De Iuliis, Antonio Marino, Marika Caruso, Sabrina Capodifoglio, Vincenzo Flati, Anna Marynuk, Valeria Marricareda, Sebastiano Ursi, Paola Lanuti, Claudio Talora, Pio Conti, Stefano Martinotti, Elena Toniato

**Affiliations:** ^1^ Unit of Predictive Medicine, SS Annunziata University Hospital of Chieti, Chieti, Italy; ^2^ Department of Biotechnological and Applied Clinical Sciences, University of L’Aquila, L’Aquila, Italy; ^3^ Odessa National Medical University, Odesa, Odessa Oblsat, Ucraina; ^4^ Department of Medicine and Aging Sciences, University G. d’Annunzio of Chieti, Chieti, Italy; ^5^ Department of Molecular Medicine, University of Rome “La Sapienza”, Rome, Italy; ^6^ Postgraduate Medical School, University of Chieti, Chieti, Italy; ^7^ Department of Medical, Oral and Biotechnological Sciences, University G. d’Annunzio of Chieti, Chieti, Italy

**Keywords:** apoptosis, autophagy, Beclin 1, EGF receptor, MAPK pathway

## Abstract

Autophagy is a not well-understood conserved mechanism activated during nutritional deprivation in order to maintain cellular homeostasis. In the present study, we investigated the correlations between autophagy, apoptosis and the MAPK pathways in melanoma cell lines. We demonstrated that during starvation the EGF receptor mediated signaling activates many proteins involved in the MAPK pathway. Our data also suggest a previously unidentified link between the EGFR and Beclin-1 in melanoma cell line. We demonstrated that, following starvation, EGFR binds and tyrosine-phosphorylates Beclin-1, suggesting that it may play a key inhibitory role in the early stage of starvation, possibly through the Beclin-1 sequestration. Furthermore, EGFR releases Beclin-1 and allows initiating steps of the autophagic process. Interestingly enough, when the EGFR pathway was blocked by anti-EGF antibodies, immunoprecipitated Beclin-1 did not bind the phospho-EGFR. In addition, an extended binding of p-Bcl2 either with Beclin-1 or with Bax was observed with a decreased activation of the stress-induced JNK kinase, thus avoiding the transduction pathways that activate autophagy and apoptosis, respectively. For this reason, we advance the hypothesis that the activation of the EGFR is a necessary event that allows the ignition and progression of the autophagic process, at least in melanoma cells.

## INTRODUCTION

Autophagy is an evolutionarily conserved pathway involved in maintaining cellular homeostasis, remodeling during the development, protection of the genome and degradation and removing of cellular materials and intracellular pathogens [1–3]. This process is conserved in different organisms, such as Dictyostelium, *C. elegans* and also in mammals [4]. Autophagy deregulation seems to be involved in several diseases [5–9]. Autophagy is characterized by the autophagosome formation within a double membrane through the interaction between a set of evolutionary conserved proteins, the ATG proteins that include Beclin-1/Atg6, LC3B/Atg8, Atg5, Atg12 and Atg13, ULK1/Atg1 [1, 10–14]. These proteins are regulated at the transcriptional and post-translational levels [15]. Autophagy is a necessary mechanism during nutritional deprivation in order to maintain cellular homeostasis and to recycle nutrients [16]. Several mechanisms seem to be involved in the activation and regulation of autophagy pathway. Specifically, in human cells the inactivation of the autophagy repressor kinase mTOR (mammalian target of rapamycin) in response to amino acid starvation contributes to autophagy through phosphorylation of ULK1/2, ATG13 and ATG14 [17]. The AMPK proteins also interact directly with components of the autophagy pathway (e.g. ULK1, Beclin-1, VPS34) in order to activate glucose starvation-induced autophagy [18–20]. In addition, previous findings suggest that activating Beclin-1 by phosphorylation modulates the way in which autophagy occurs [21–22]. Furthermore, substantial evidence suggests that dissociation of Bcl-2 from Beclin-1 may also be an important mechanism for activating autophagy in response to starvation [23]. In fact, the activity of the Beclin-1/VPS34 autophagy complex seems to be inhibited by the direct binding of Beclin-1 to Bcl-2 [24]. Furthermore, a study of Wei *et al*. demonstrated that the JNK1-mediated phosphorylation of Bcl-2 activates the starvation-induced autophagy through the dissociation of Bcl-2 from Beclin-1 [25].

Moreover, multiple signaling cascades, including the Extracellular signal Regulated Kinase (ERK), a protein that plays a pivotal role in various biological processes of cell physiology, such as proliferation, have been shown to regulate autophagy [26, 27]. Several findings demonstrated that the ERK cascade components display increased association with ATG5–ATG12-positive pre-autophagosomal structures and with lipidated ATG8 family proteins, such as LC3-II and GABARAP12, following growth factor stimulation. At the same time, autophagy proteins seem to regulate ERK phosphorylation as an unconventional function of the ATG7/ATG5– ATG12/LC3-II, independently of their canonical role in lysosomal proteolysis [28]. However, the signaling mechanisms that activate the autophagy-essential protein complexes, in particular Beclin-1 and its protein-protein interactions, are not well understood.

In the present study, we investigated the correlations between autophagy, apoptosis and proliferative pathways in melanoma cell lines. We found, for the first time in melanoma cell line, a previously described [29–30] interaction between the MAPK mediated pathway and the proteins involved in the autophagosome formation, mainly the p70S6 kinase, LC3B and Beclin-1. We also investigated the interactions between Beclin-1, Bcl-2 and Bax, a protein complex that modulates the autophagy/apoptosis switch. Our data suggest a link between the EGFR and Beclin-1, previously studied in other models of cancer cell line [31–33]. These findings showed that the EGF receptor (EGFR) mediated signaling is tightly correlated to the phosphorylation of Beclin-1 and leading to the modulation of first step in the autophagy initiation process.

## RESULTS

### M14 and 793 cell viability

**Figure 1:**
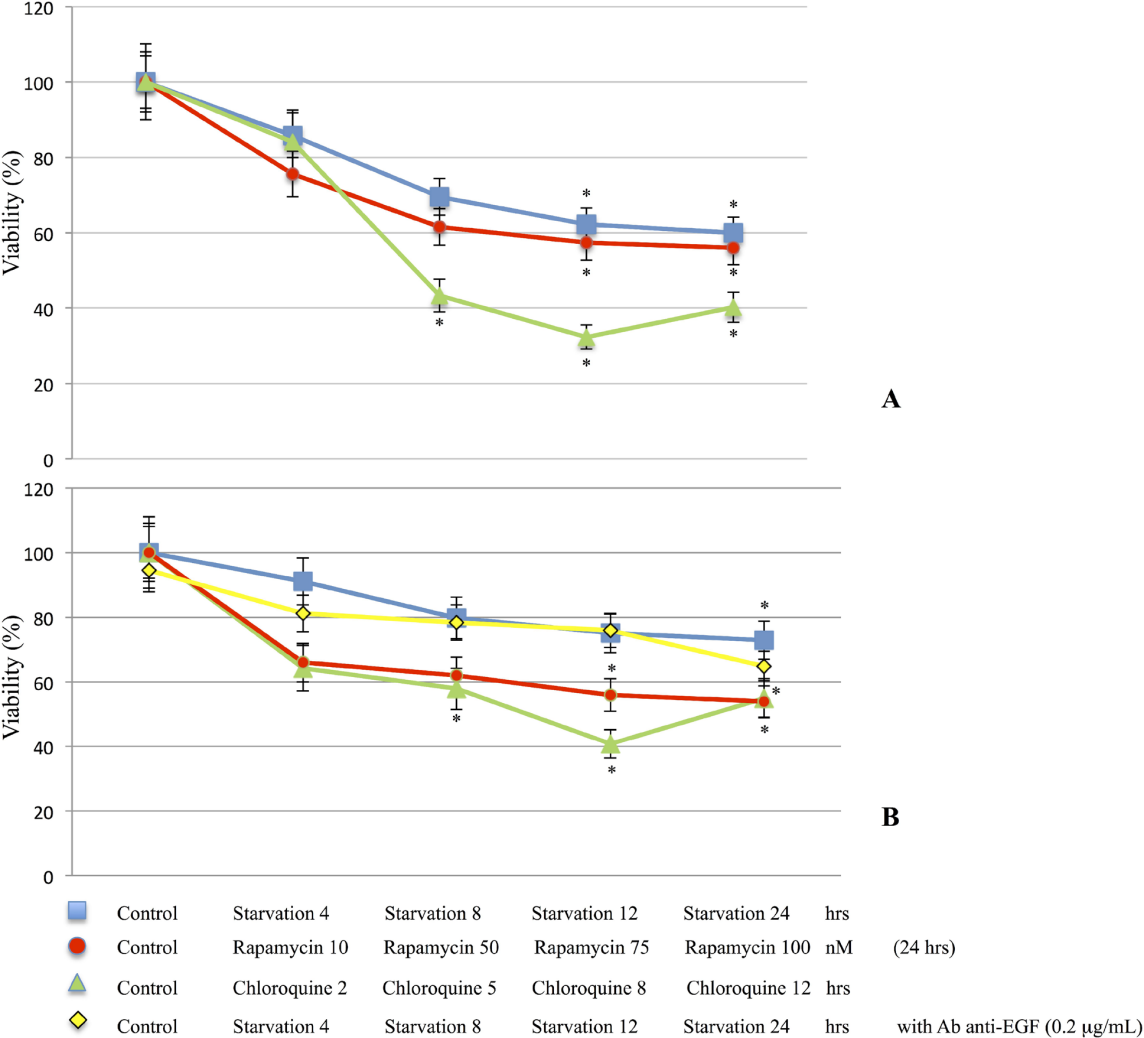
Cell viability determination M14 (**A**) and 793 (**B**) cell lines were starved for 4, 8, 12 and 24 hrs or treated with Rapamycin for 24 hrs at different concentrations (10, 50, 75 and 100 nM), Chloroquine 10 nM for 2, 5, 8 and 12 hrs. In addition, 793 cells were starved and treated with anti-EGF antibody 0.2 μg/mL. M14 and 793 cells undergoing starvation, Rapamycin or Chloroquine treatments showed a significant decrease of viability compared to the control group. M14 cell line, (A). Control *vs* Starvation groups: ANOVA *p* = 0.012 [Control *vs* Starvation 12 hrs/Starvation 24 hrs: *p <* 0.05]; Control *vs* Rapamycin groups: ANOVA *p* = 0.007 [Control *vs* Rapamycin 75 nM/Rapamycin 100 nM: *p <* 0.05]; Control *vs* Chloroquine groups: ANOVA *p <* 0.001 [Control *vs* Chloroquine 5 hrs/Chloroquine 8 hrs/Chloroquine 12 hrs: *p <* 0.05]. 793 cell line, (B). Control *vs* Starvation groups: ANOVA *p* = 0.027 [Control *vs* Starvation 24 hrs: *p <* 0.05]; Control *vs* Rapamycin groups: ANOVA *p* = 0.012 [Control *vs* Rapamycin 75 nM/Rapamycin 100 nM: *p <* 0.05]; Control *vs* Chloroquine groups: ANOVA *p* = 0.004 [Control *vs* Chloroquine 5 hrs/Chloroquine 8 hrs/Chloroquine 12 hrs: *p <* 0.05]. In addition, 793 cells treated with anti-EGF antibody, with or w/out Starvation medium, did not show significant differences (B). ^*^p < 0.05.

The proliferation of M14 and 793 cells was measured using the MTT assay. Cell viability of starved or treated cells was compared to the control group and expressed as a ratio of color intensity. As shown in Figure 1, M14 (1A) and 793 (1B) cells undergoing starvation, Rapamycin or Chloroquine treatments showed a significant decrease of viability when compared to controls. In addition, 793 cells treated with anti-EGF antibody, with or without starvation medium, showed no significant differences.

### Evaluation of autophagosome formation during starvation or Rapamycin treatment

**Figure 2:**
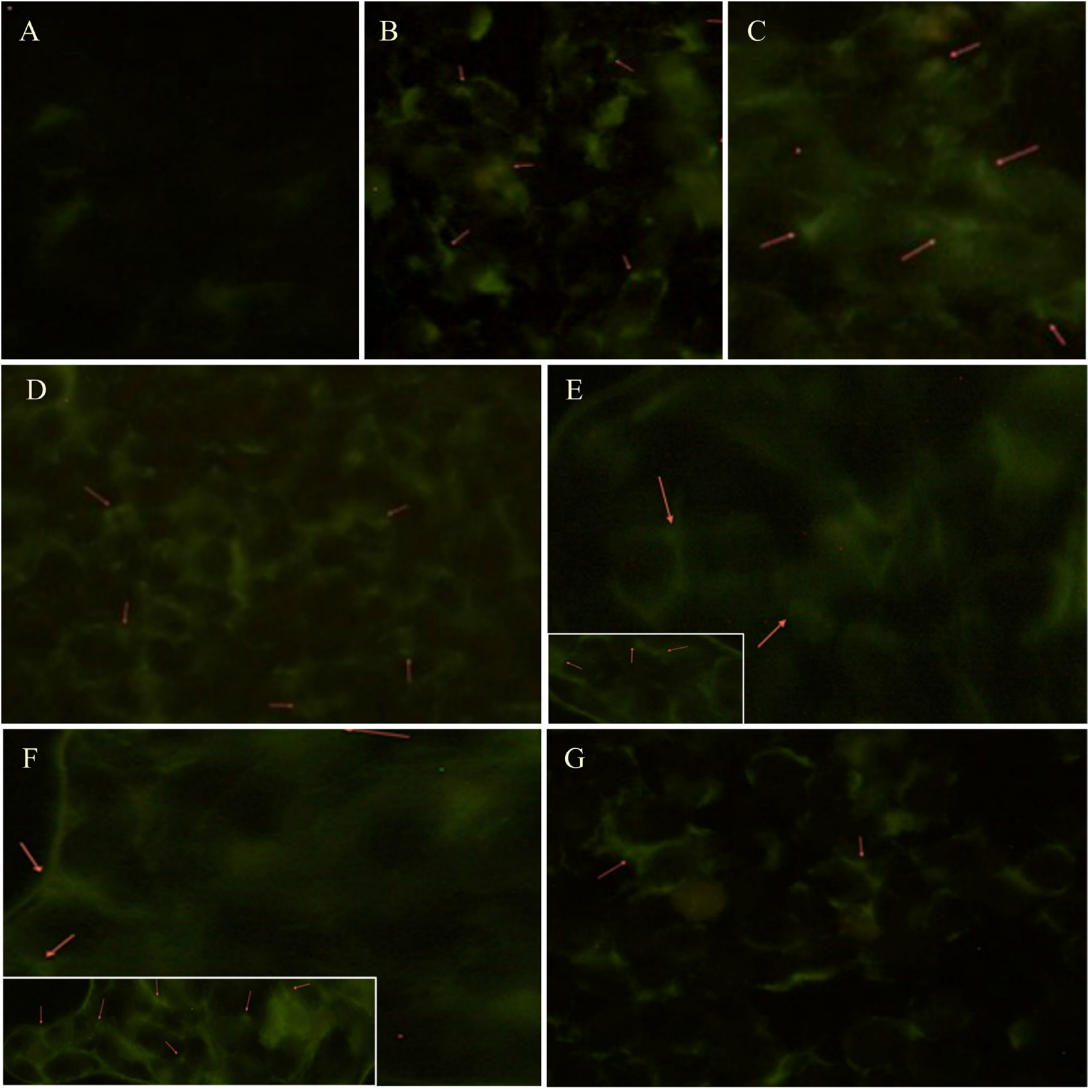
Autophagosomes formation analysis during starvation or Rapamycin treatment Autophagy was determined by fluorescence microscopic detection of autophagosomes formation using the Monodansylcadaverine (0.05 mmol/l MDC) labeling assay. These pictures evidence an increased presence of autophagosomes (red arrows) in M14 cells either undergoing starvation for 4 hrs (**B**), 12 hrs (**C**), 24 hrs (**D**) or treated with 10 (E) and 50 (**F**; inset: detail of autophagosomes formation) nm of Rapamycin compared to control group (**A**). Moreover, cells treated with 100 nM Rapamycin (**G**) seem to be undergoing apoptotic processes.

Autophagosome formation was analyzed using the Monodansylcadaverine (MDC) labeling assay. Figure 2 evidenced an increased presence of autophagosomes in M14 cells undergoing either starvation or treatments with Rapamycin at 10 and 50 nM compared to control group. Moreover, cells treated with 100 nM Rapamycin seemed to be undergoing apoptotic processes. Furthermore, the 793 cell line evidenced similar findings (Supplementary Figure 1).

### Late starvation induces EGF but not VEGF release

**Figure 3:**
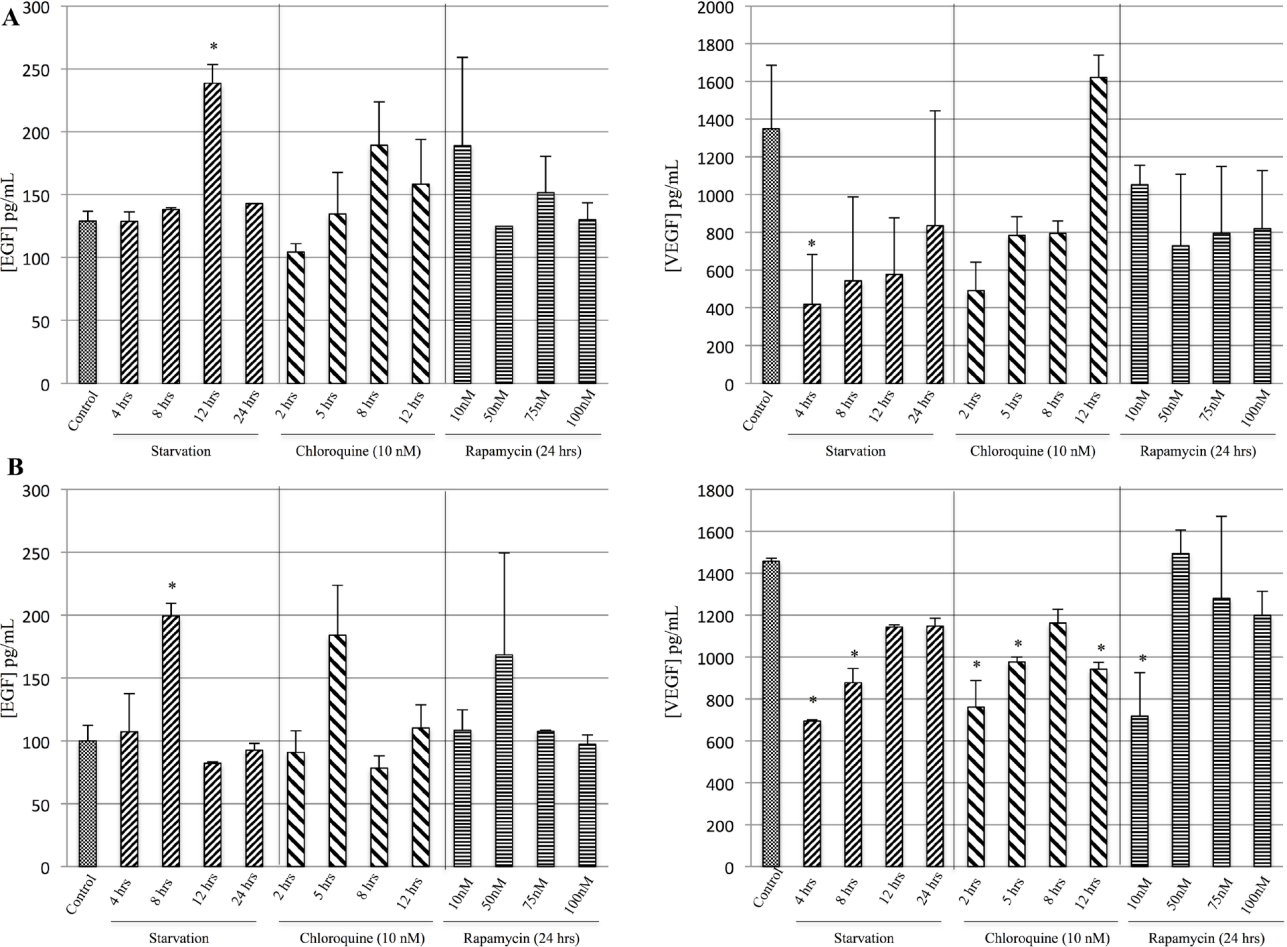
Evaluation of EGF and VEGF release during starvation The concentration of EGF and VEGF released in the supernatants was determined using enzyme immunoassays. M14 cells (**A**) starved for 12 and 4 hrs showed a significant increase of EGF and decrease of VEGF release as compared to the control group, respectively. Furthermore, 793 cell line (**B**) starved for 8 hrs showed a significant increase of EGF as compared to controls. In addition, 793 cells starved for 4 and 8 hrs of starvation, or treated with 2, 5 and 12 hrs of Chloroquine or with 10 nM Rapamycin evidenced a significant decrease in VEGF compared to the control group. ^*^p < 0.05.

EGF and VEGF release on cells treated with starvation, Rapamycin or Chloroquine was determined using enzyme immunoassays. In particular, M14 cell line undergoing starvation for 4 and 12 hrs showed a decrease of VEGF release and a significant increase on EGF as compared to the control group, respectively (Figure 3A). Furthermore, the 793 cells starved for 8 hrs showed a significant rise in EGF release as compared to controls (Figure 3B). In addition, the 793 cells starved for 4 and 8 hrs or treated with Chloroquine for 2, 5 and 12 hrs or with 10 nM Rapamycin evidenced a significant reduction of VEGF compared to the control group (Figure 3B).

### p-ERK expression induced by starvation on M14 and 793 cell lines

**Figure 4:**
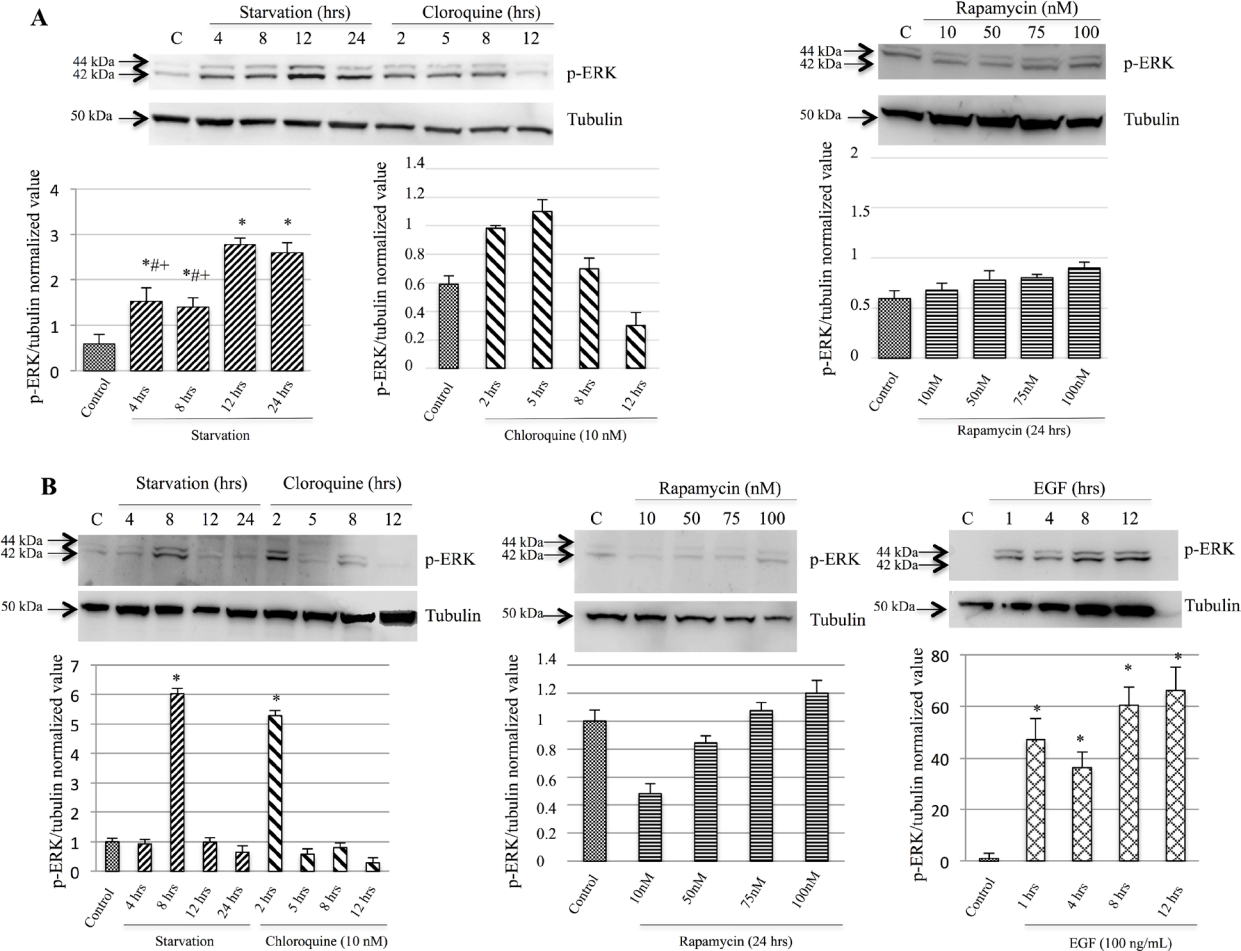
p-ERK protein expression was induced during starvation p-ERK protein expression was evaluated by Western blot analysis. M14 cells (**A**) starved for 4, 8, 12 and 24 hrs showed a statistically significant difference compared to the control group. These results evidenced a significant increase of p-ERK expression in cells starved for 12 and 24 hrs when compared with 4 and 8 hrs of the starvation groups. Western blot analysis did not show a modulation in M14 cells treated with either Chloroquine at different times of exposure or Rapamycin at different concentrations. 793 cell line (**B**) showed a significant increase of p-ERK expression in cells undergoing 8 hrs of starvation or treated with 2-hrs Chloroquine compared to controls. Western blot analysis did not show a modulation in 793 cells treated with different concentrations of Rapamycin. Moreover, 793 cells treated with EGF showed a significant increase of p-ERK expression comparable to the control group, in a time-independent manner. Time of exposure of p-ERK and tubulin were 12.9 and 4.3 s, respectively. ^*^p < 0.05 as compared to controls; ^#^*p <* 0.05 as compared to 12-hrs starvation; ^+^*p <* 0.05 as compared to 24-hrs starvation.

p-ERK protein expression was evaluated by Western blot analysis. Specifically, as shown in Figure 4A, M14 cells undergoing starvation evidenced a significant increase of p-ERK expression compared to control group. Western blot analysis show a slight p-ERK modulation in M14 cells treated with Chloroquine at different times of exposure. However, such modulation was not observed after Rapamycin treatment, wich blocks the mTOR kinase activity. Moreover, as depicted in Figure 4B, comparable to the M14 cell line the 793 showed a more significant modulation of p-ERK expression when undergoing 8 hrs of starvation or 2 hrs Chloroquine treatment compared to control group, wich could be related to the physiological property of the cell line. Western blot analysis did not show any significant modulation of p-ERK in 793 cells treated with Rapamycin at different concentrations. In addition, as a positive control, the 793 cells treated with EGF showed a significant increase of p-ERK expression, compared to the control group, in a time-independent manner.

### p-p70/85 S6 Kinase expression induced by starvation on M14 cell line

**Figure 5:**
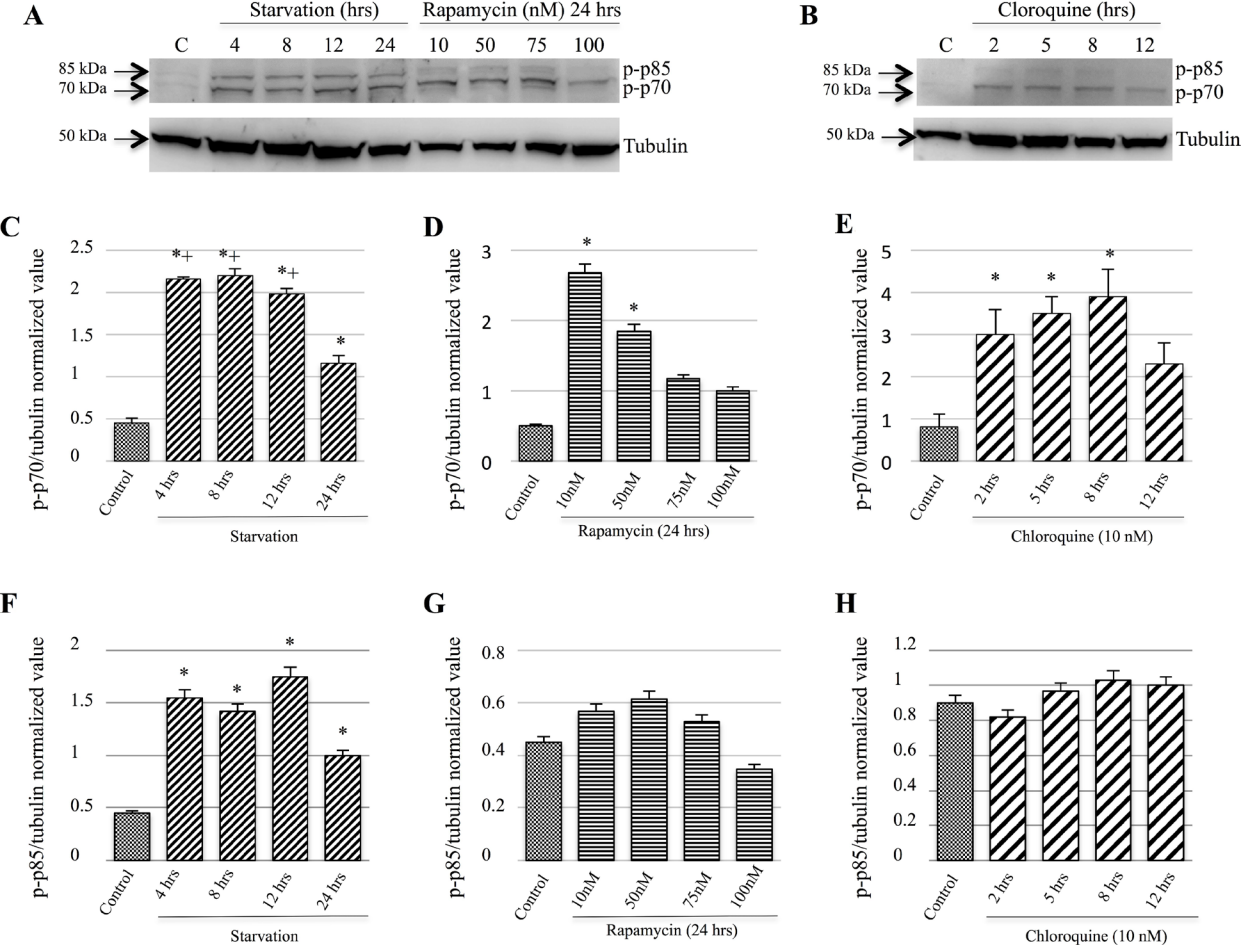
p-p70 and p-p85 S6 Kinase proteins expression were induced during starvation p-p70 and p-p85 S6 Kinase proteins expression were evaluated by Western blot analysis (**A**, **B**). M14 cells starved for 4, 8, 12 and 24 hrs showed statistically significant differences compared to the control group (**C**). The same results were observed among the 24-hrs starvation group and the 4, 8 and 12 hrs groups (C). This Figure also evidences a significant increase of p-p70 expression in cells treated with 10 and 50 nM Rapamycin compared to the controls (**D**). Furthermore, Western blot analysis showed a significant difference between controls and cells treated with Chloroquine for 2, 5, 8 hrs (**E**). As for as concern p-p85 protein expression, a significant increase among the starvations groups was observed as compared to the controls (**F**). These findings were not observed when the M14 cells were treated with Rapamycin (**G**) or Chloroquine (**H**). Time of exposure of p-70/85 and tubulin were 13 and 4.3 s, respectively. ^*^p < 0.05 as compared to controls; ^+^*p <* 0.05 as compared to 24-hrs starvation.

With regard to the M14 cell line, Western blot analysis (Figure 5A, 5B) showed a significant increase of p-p70/85 S6 Kinase levels with a higher expression of the p-p85 isoform, mostly detectable on the starved cell line (Figure 5F–5H). As far as concern the p-p70 isoform, we found an increase of protein phosphorylation either on starved or Rapamycin and Chloroquine treated cells without any marked differences (Figure 5C–5E). Moreover, the 793 cell line evidenced similar findings (Supplementary Figure 2).

### Autophagy process evaluation by LC3BII expression analysis

**Figure 6:**
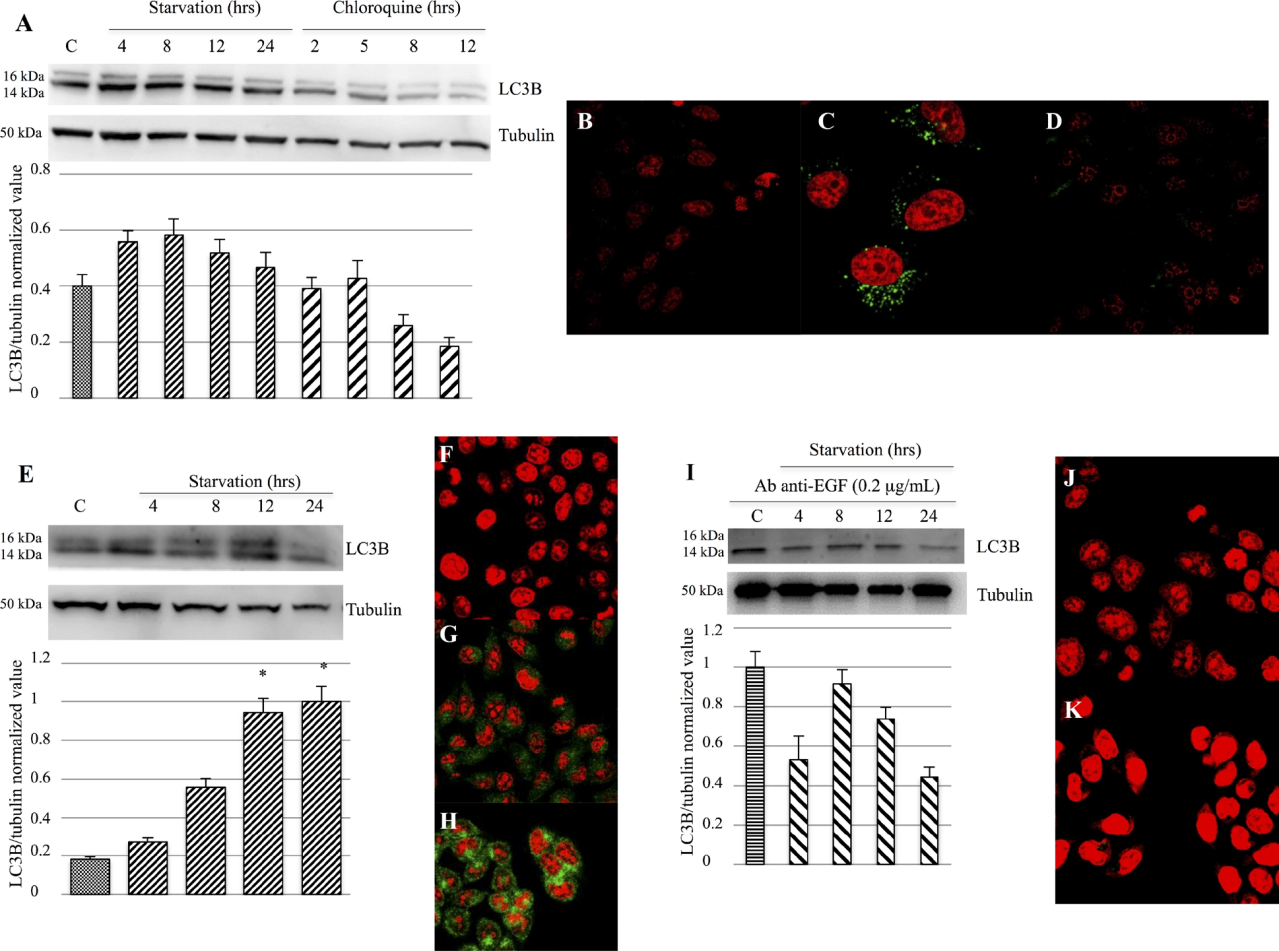
Evaluation of LC3B activation and expression (**A**–**D**) Western blot analysis (A) did not show a prominent LC3B modulation in M14 cells, but only a slight increase in cells undergoing starvation or treated with Chloroquine for 2 or 5 hrs, as compared to the control group. Confocal microscopy showed a significant LC3B expression in M14 cells treated with 12-hrs Chloroquine (C) or starved for 12 hrs (D), compared to the control group (B). (**E**–**H**) Western blot analysis (E) evidenced a strong LC3BII modulation in 793 cells. In particular, a significant rise in LC3BII expression was evident on cells starved for 12 and 24 hrs, compared to the control group. These findings were confirmed by confocal microscopy, that showed a significantly LC3BII increase in 793 cells starved for 12 hrs (H) or treated with 2-hrs Chloroquine (G), compared to the control group (F). (**I**–**K**) Western blot analysis (I) did not show an LC3B up-regulation in 793 cells starved and treated with anti-EGF antibody 0.2 μg/mL. These findings were confirmed by confocal microscopy (J: Control group treated with anti-EGF antibody; K: 793 cell undergoing 12 hrs of starvation and anti-EGF antibody treatment). Time of exposure of LC3B and tubulin were 8.2 and 4.3 s, respectively. ^*^p < 0.05 as compared to control group.

Western blot analysis and confocal microscopy were performed in order to evaluate LC3BII expression. Our findings did not reveal a LC3BII prominent modulation in M14 cells, but only a slightly increase in cells undergoing starvation or treated with 2 or 5 hrs of Chloroquine compared to the control group (Figure 6A). Furthermore, cells treated with Chloroquine for 8 and 12 hrs showed an LC3BII decreased expression (Figure 6A). Confocal microscopy analysis evidenced an increase of LC3B expression in M14 cells treated with 2 hrs Chloroquine (Figure 6C) and when undergoing 12 hrs of starvation (Figure 6D) compared to the control group (Figure 6B). Furthermore, we observed an LC3BII strong modulation in 793 cells. In particular, a significantly increased LC3BII expression was evident on cells starved for 12 and 24 hrs, compared to the control group (Figure 6E). These findings were confirmed by confocal microscopy, that showed a significant LC3BII rise in 793 cells undergoing 12 hrs of starvation (Figure 6H) or 2 hrs Chloroquine treatment (Figure 6G), compared to the control group (Figure 6F). Moreover, the 793 cell line starved and treated with anti-EGF antibody did not show an LC3BII up-regulation (Figure 6I), as confirmed by confocal microscopy (Figure 6J and 6K).

### Anti-EGF antibody reduced apoptotic processes

**Figure 7:**
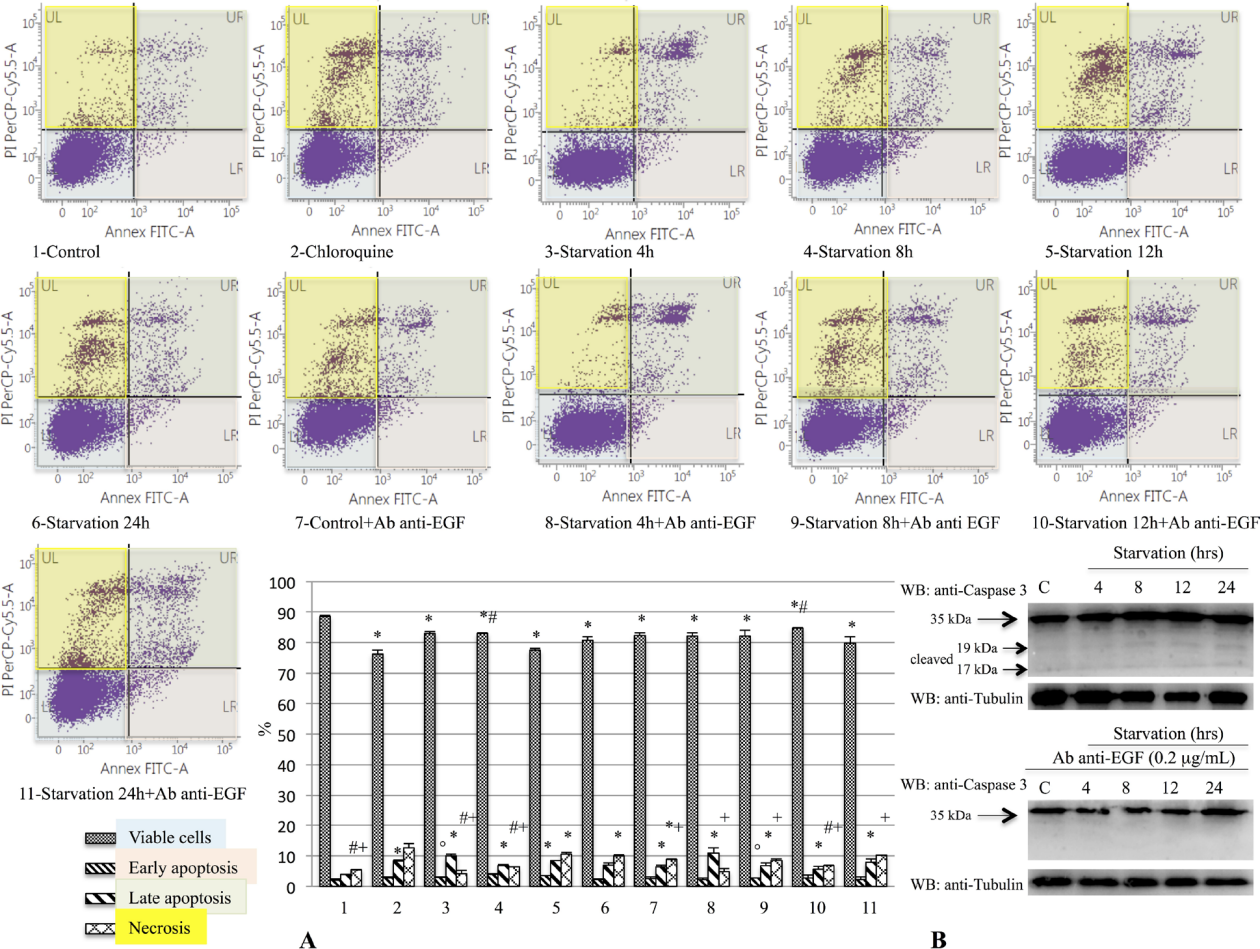
Evaluation of early and late apoptosis on 793 cells starved with or without anti-EGF antibody The induction of apoptotic processes was analyzed by caspase-3 expression analysis and by Annexin assay using Western blot analysis and Flow Cytometry, respectively. This figure shows a decrease of viable cell percentages on samples starved with or w/out anti-EGF antibody compared to the control group (**A**, ANOVA: *p <* 0.001). We also demonstrated a significant difference for this parameter between samples starved for 12 hrs with and without anti-EGF antibody (A, ANOVA: *p <* 0.001). Furthermore, early apoptosis was more prominent on 793 cells starved for 8 hrs, in respect to cells starved for the same time and treated with anti-EGF antibody and on cells starved for 8 and 12 hrs compared to 4 hrs and to the control group, respectively (A, ANOVA: *p <* 0.001). With regard to late apoptosis, this picture shows a significant difference between control groups with and w/out anti-EGF antibody and on cells starved with and w/out anti-EGF antibody in respect to the control group (A, ANOVA: *p <* 0.001). Finally, we demonstrated a significant increase of necrosis on cells treated with Chloroquine. In addition, we evidenced a necrotic process more evident on 12-hrs starved cells as compared either to the same treatment with anti-EGF antibody, or to control and 4- and 8-hrs starved groups (A, ANOVA: *p <* 0.001). Caspase-3 cleaved expression (**B**) was modulated on 793 cells starved for several different times, especially for 12 and 24 hrs, but not on cells treated with starvation and anti-EGF antibody, confirming Flow Cytometry data. Time of exposure of Caspase-3 and tubulin were 4.3 s for both. ^*^p < 0.05 compared to control group; ^#^*p <* 0.05 compared to 12-hrs starved group; °: *p <* 0.05 compared to 8-hrs starved group; ^+^*p <* 0.05 compared to Chloroquine group.

The induction of apoptotic processes was evaluated by the caspase-3 expression analysis and by Annexin assay using Western blot analysis and flow cytometry, respectively. Our findings showed a decrease of viable cell percentage on starved samples with and w/out anti-EGF antibody as compared to the control group (Figure 7A). Moreover, we evidenced a significant difference of viability between samples undergoing 12 hrs of starvation with and without anti-EGF antibody (Figure 7A). Furthermore, early apoptosis was more prominent on 793 cells starved for 8 hrs, compared to cells starved for the same time but also treated with anti-EGF antibody and on 8- and 12-hrs starvation groups, compared to 4-hrs and to the control groups, respectively (Figure 7A). Concerning the late apoptosis, in Figure 7A is shown a significant difference between control groups with and w/out anti-EGF antibody. In addition, we observed a more pronounced late apoptosis in cells undergoing starvation with and w/out anti-EGF antibody in respect to the control group (Figure 7A). Finally, we observed a significant difference between the levels of necrosis in all the studied groups when compared to the 793 cells treated with Chloroquine. The necrotic process was more prominent in 12-hrs starved cells compared either to the same treatment but in presence of anti-EGF antibody, or to control and to the 4- and 8-hrs starved groups (Figure 7A). Furthermore, as shown in Figure 7B, Flow Cytometry data were confirmed by caspase-3 cleaved expression, that was modulated on 793 cells starved for several different times, especially for 12 and 24 hrs, but not in cells undergoing starvation and treated with anti-EGF antibody. Moreover, similar findings regarding the caspase-3 cleaved expression were observed on M14 cell line (Supplementary Figure 3).

### EGFR phosphorylation is required for the autophagy activation and regulation

**Figure 8:**
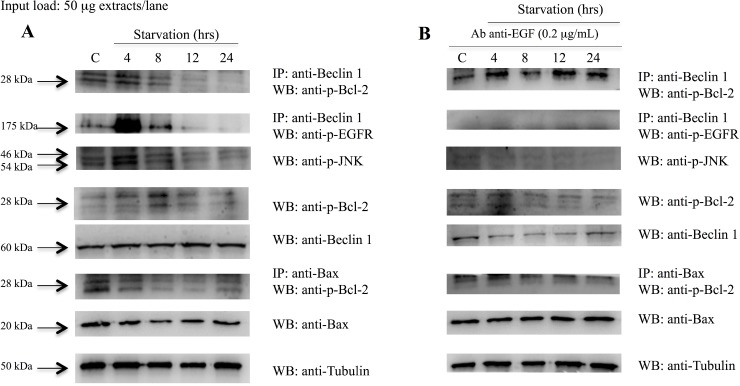
Analysis of Beclin 1 protein interactions and Bcl2 and Bax protein expression on 793 cells starved with and without anti-EGF antibody Western blot in (**A**), shows that: phospho-EGFR strongly binds Beclin 1 on 793 cells starved for 4 hrs; p-Bcl2 bind Beclin 1 up to 4 hrs of starvation and then detach; p-JNK and p-Bcl2 protein expressions increase at 4 and 8 hrs of starvation, respectively; Bcl2 and Bax were attached until 8 hrs of starvation and, afterwards, they were detached. (**B**) 793 cells starved and treated with anti-EGF antibody showed that: Beclin 1 and p-EGFR were detached; Beclin 1 and p-Bcl2 stayed attached during starvation; p-JNK and p-Bcl-2 expression levels were constantly down-regulated; p-Bcl2 and Bax were constantly attached. Time of exposure of p-Bcl2, p-EGFR, p-JNK, Beclin-1, Bax and tubulin were 7.5, 2, 15, 9.6, 5 and 4.3 s, respectively.

In order to investigate whether EGFR plays a pivotal role in the modulation of the autophagic pathway, Beclin-1 immunoprecipitation and phospho-EGFR western blots were performed. Figure 8A shows that phospho-EGFR strongly binds Beclin 1 at 4 hrs of starvation and, thus, it could participate in Beclin 1 phosphorylation. Beclin-1 phosphorylation by phospho-EGFR decreased after 4 hrs of starvation. Thus, we postulate that EGFR play a role in the inhibition of the autophagy via the Beclin-1 sequestration in the early starvation. Subsequently, the activated form of the EGFR, dimerizes, activates and releases Beclin 1 thus allowing initiation of the autophagic process. Beclin-1 immunoprecipitation and phopsho-Bcl-2 blot demonstrated that p-Bcl-2 binds Beclin-1 up to 4 hrs of starvation, then it detaches. These data were confirmed by p-JNK and p-Bcl-2 blots, which showed a significant increase of these protein expressions at 4 and 8 hrs of starvation, respectively, suggesting that Bcl-2 was detached from Beclin-1 by p-JNK phosphorylation. In order to better understand the shift from autophagy to apoptosis, Bax immunoprecipitation and phospho-Bcl-2 blot were performed. This experiment showed that Bcl-2 and Bax were attached up to 8 hrs of starvation and, afterwards, they were detached thus allowing the start of the apoptotic pathway.

Summarising, when 793 cells were starved and treated with anti-EGF antibody (Figure 8B) we observed that: i) Beclin-1 and p-EGFR were detached; ii) Beclin-1 and p-Bcl-2 stayed attached during starvation; iii) the p-Bcl-2 and p-JNK expression levels were constantly down-regulated; iv) p-Bcl-2 and Bax were attached and this binding seemed to not allow the onset of apoptosis. These findings demonstrated that, when the EGFR was blocked by anti-EGF antibody, the autophagic and apoptotic processes seemed to be delayed. For this reason, we hypothesize that the activation of the EGFR is a necessary condition in order to begin the autophagy.

### Modulation of the EGF gene transcription during starvation

**Figure 9:**
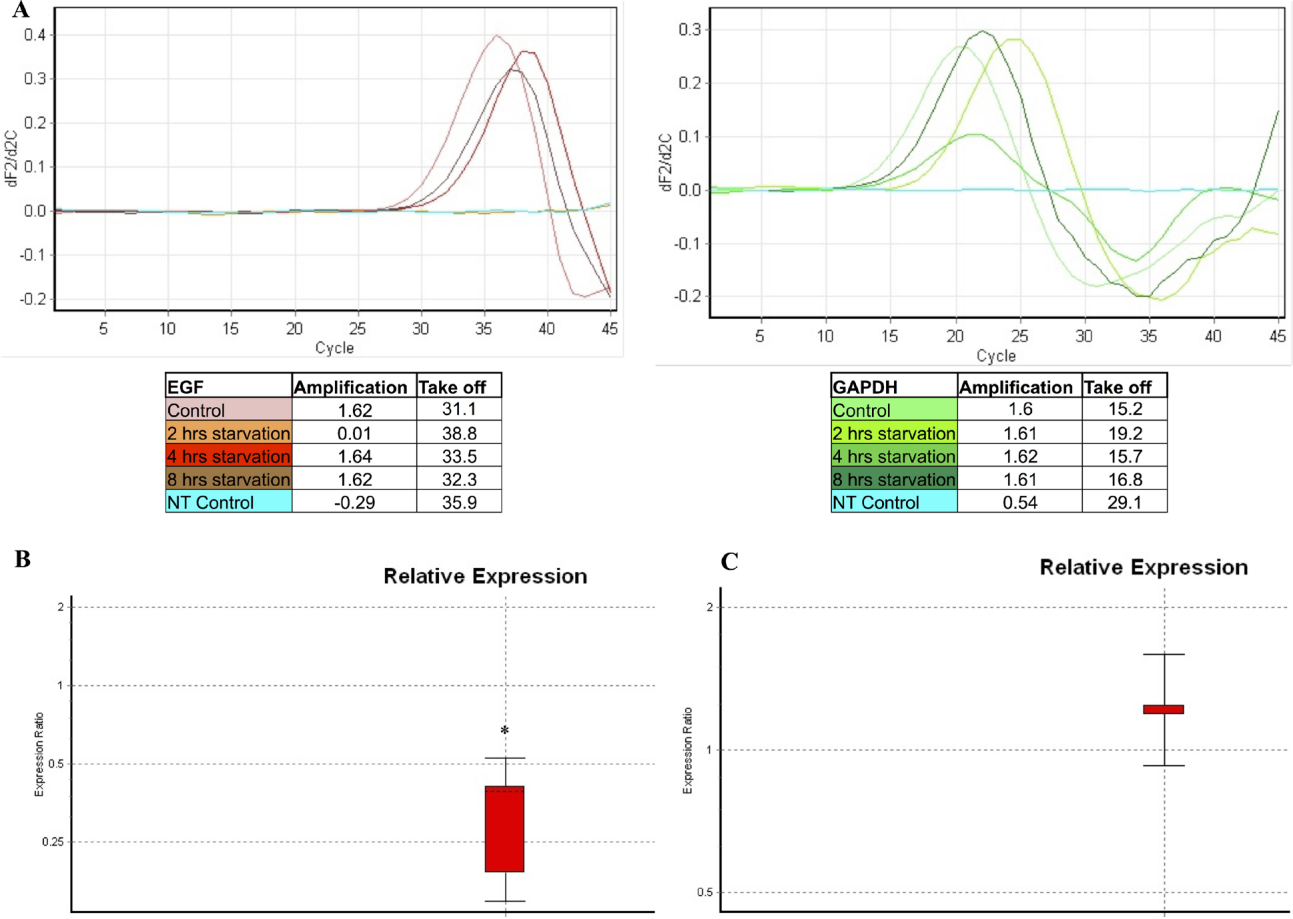
EGF gene transcription analysis evaluated in starved samples A qPCR was performed in order to evaluate the EGF transcription in samples starved for 2, 4 and 8 hrs, as compared to the control group. The GAPDH gene transcription was used as a reference gene. This picture represents a single set of experiments. (**A**) The EGF transcription was absent at 2 hrs of starvation. Subsequently, a lower EGF transcription was observed at 4 hrs of starvation compared to the control groups. Finally, the EGF transcription returned to values similar to the control group starting from 8 hrs of starvation. (**B**) Comparative analysis of EGF relative expression on samples starved for 4 hrs compared to the control group. (**C**) Comparative analysis of EGF relative expression on samples starved for 8 hrs compared to the control group. NT Control: no template control. ^*^: *p* = 0.005.

The EGF transcription and the relative ratios between controls and samples starved for 2, 4 and 8 hrs were evaluated. As shown in Figure 9A, EGF was not transcribed after 2 hrs of starvation. In fact, the target gene amplification was lower than 0.5, whereas the amplification of the reference gene (GAPDH) was higher than 1.5. Furthermore, a significantly lower EGF transcription was observed after 4 hrs of starvation as compared to the control group (Figure 9B, ratio: 0.29 ± 0.13, *p* = 0.005). In addition, samples starved for 8 hrs showed an EGF transcription level similar to controls (Figure 9C, ratio: 1.2 ±0.02, *p* > 0.05). Finally, starved cells treated with anti-EGF antibody did not show any modulation of the EGF gene transcription (data not shown).

## DISCUSSION

**Figure 10:**
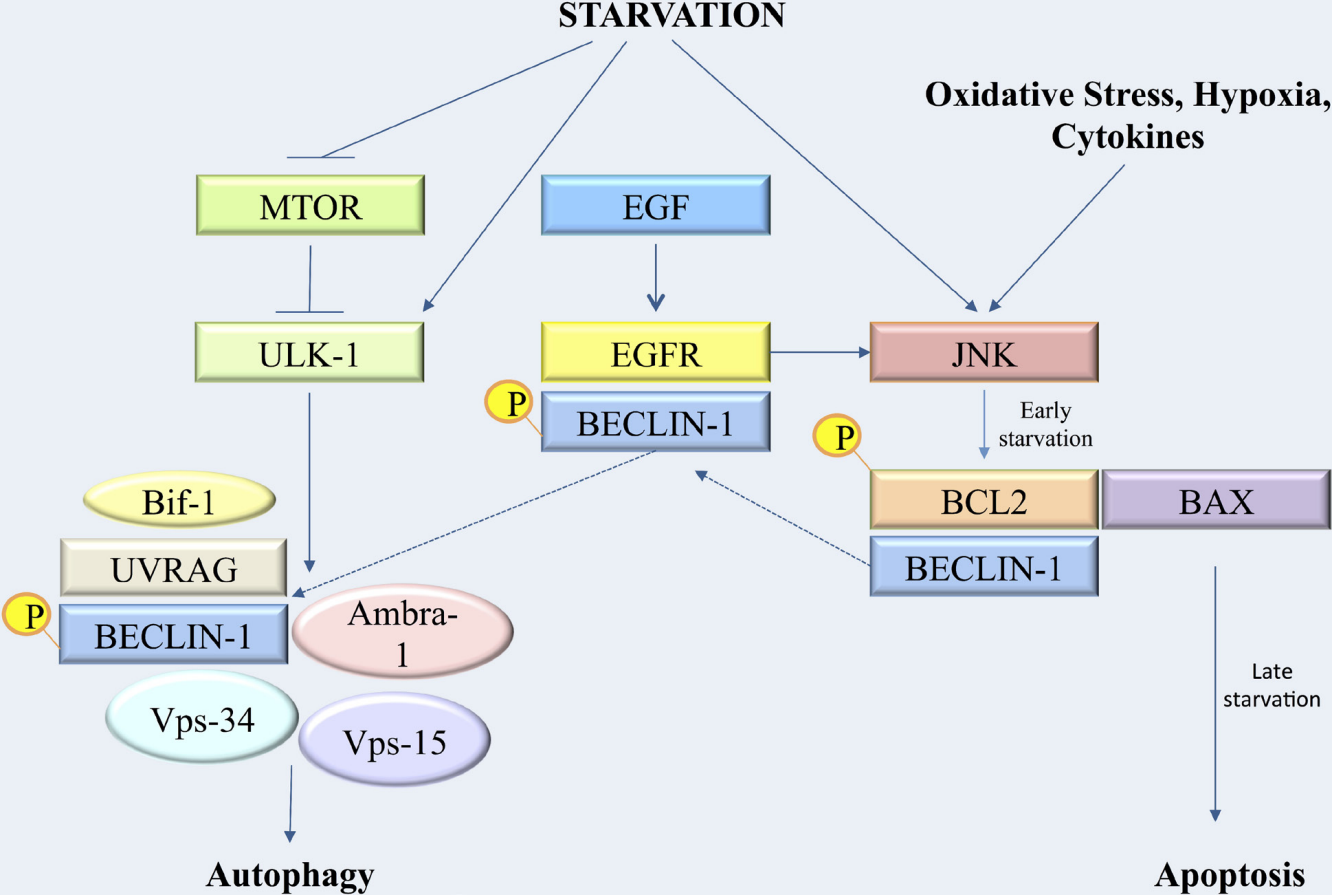
Synoptic figure recapitulating the cascade of metabolic events leading to autophagy regulation and transition to apoptosis after starvation

Autophagy is a key process involved in cellular homeostasis and in the survival response during nutrient deprivation [1–3]. The interaction between a set of evolutionary conserved proteins, the ATG proteins (e.g. Beclin 1/Atg6, LC3B/Atg8, Atg5, Atg12 and Atg13, ULK1/Atg1) leads to autophagosome formation [1, 10–14]. Several pathways seem to be involved in autophagy activation and most of them involve the inhibition of mTORC1 [17]. On the contrary, in many cases, the negative modulation of autophagy involves Beclin 1 inhibition by the binding with Bcl-2 [23, 24]. Furthermore, MK2 and MK3 kinases, which take a part in the MAPK pathway, seem to be crucial for starvation-induced autophagy by Beclin-1 S90 phosphorylation [34]. The aim of the present study was to better understand the mechanisms and the pathway underlying autophagy initiation in melanoma cells after starvation. We identified a direct link between the MAPK pathway and the autophagy protein Beclin-1. In fact, our results demonstrated that during starvation the EGFR mediated the phosphorylation of other proteins involved in the MAPK pathway, such as Erk. As previously described, the activation of this pathway led to the phosphorylation of an autophagy inhibitor, Bcl-2, that resulted in the disruption of its binding with Beclin-1 [25]. Our data suggest a previously unidentified link between the EGFR and Beclin-1 in melanoma cell line. In fact, we demonstrated that the EGFR play a key role in the early starvation, acting as an autophagy inhibitor promoter, through a direct interaction with Beclin-1. So, after EGF binding to its receptor, EGFR autophosphorylates and directly tyrosine-phosphorylates Beclin-1 as a necessary event for its further release and initiation of the autophagic process (Figure 8A). As shown, Beclin-1 and p-EGFR complex coimmunoprecipitates following few hours after induction of starvation. Furthermore, the complex starts to dissociate later on and Beclin-1 move for activating pathways involved in the autophagosome formation. In order to better characterize the role of the EGF signaling we also investigated the EGF transcription level, that represent an autocrine stimuli by the cancel cell line, by using a qPCR. We evidenced a down-regulation of the EGF gene expression after 2 hrs of starvation, and a slight increase of EGF expression starting at 4 hrs of starvation, but characterized by transcription levels lower than the control group. This EGF modulation in the early hours of starvation seems to function as a signal for the EGFR in order to strongly phosphorylate and release Beclin-1. In addition, the absence of the EGF transcription modulation through an anti-EGF antibody treatment (data not shown), does not result in EGFR binding and phosphorylation of Beclin-1. In order to analyze another growth factor, which is mostly involved in tumor progression we performed ELISA experiments measuring VEGF release from melanoma cell line undergoing starvation. As shown on Figure 4, on a comparative analysis on EGF induction, we gained opposite results having a decrease expression of VEGF within the first hours following starvation induction. These findings highlight the peculiar and unique role of EGF on priming the tyrosine phosphorylation-related activation of EGF receptor closely linked to Beclin 1 maturation for the autophagy signaling. Furthermore, we evidenced an increase of p-Bcl-2 protein level after 8 hrs of starvation. These findings were confirmed by a raise of LC3BII level starting at 8 hrs since starvation, as demonstrated either in Western analysis or in the confocal microscopy experiments. In addition, relating to the shift between autophagy and apoptosis, we showed, as expected, that Bcl-2 and Bax remained complexed up to 8 hrs since starvation and, afterwards, they dissociate in order to activate the apoptotic pathway. In fact, the Annexin assay and the cleaved Caspase-3 expression level demonstrated a more prominent apoptosis at 12 hrs of starvation. Interestingly, when the EGFR pathway was blocked by an anti-EGF antibody, the 793 cells seemed to be in a particular state characterized by a Beclin-1 and p-EGFR detachment, an extended binding of p-Bcl-2 either with Beclin-1 and Bax, which resulted in a p-Bcl-2 protein down-regulation. These findings could demonstrate a delayed activation of the autophagy and of the apoptosis processes. For this reason, as recapitulated on Figure 10, we hypothesize that the activation of the EGFR, even though inhibiting autophagy in the early starvation, is a necessary condition in order to initiate the autophagy process by binding and phosphorylating Beclin-1. Further studies will be required and warranted to completely outline the mechanisms involved in autophagy in our experimental setup, especially to better understand the switch between the autophagic and apoptotic processes in order to leverage them as possible novel cancer therapeutic approaches.

## MATERIALS AND METHODS

### Cell culture

M14 and 793 melanoma cell lines were purchased from ATCC. M14 and 793 cell lines were grown in DMEM and RPMI 1640, respectively, supplemented with 10% FBS, 2 mM L-glutamine and 1% Pen-Strep at 37° C and 5% CO_2_. The cells were starved for the indicated time period (4, 8, 12 and 24 hrs) by culturing in DMEM or RPMI 1640 supplemented with 0.1% FBS, 2 mM L-glutamine and 1% Pen-Strep at 37° C and 5% CO_2_. Where indicated, the cells were treated with Rapamycin for 24 hrs at different concentrations (10, 50, 75 and 100 nM), Chloroquine 10 nM for 2, 5, 8 and 12 hrs as indicated, with EGF 100 ng/mL or anti-EGF antibody 0.2 μg/mL. Rapamycin, Chloroquine and EGF were purchased from Sigma-Aldrich (Saint Louis, MO, USA). The anti-EGF antibody was purchased from Abcam (Cambridge, UK).

### Cell viability

The cell viability was measured with 3-(4,5-dimethylthiazol-2-yl)-2,5-dipenyl tetrazolium bromide (MTT) (Sigma-Aldrich, Saint Louis, MO, USA) assay according to standard procedure for adherent cells. At this point, the absorbance (optical density) was measured at 530 nm using a micro-plate reader (Infinite F50, TECAN). The experiment was performed in triplicate.

### Apoptosis detection

Apoptosis analysis by Flow Cytometry was performed according to the manufacturer's instructions (Trevigen, Gaithersburg, MD, USA). A double labeling panel, Annexin V-FITC and Propidium Iodide, was used in order to better differentiate between early and apoptotic/necrotic events, respectively. The experiment was performed in triplicate.

### Protein extraction and Western blot

Total proteins were extracted from lymphomonocyte in lysis buffer (50 mM Tris-HCl pH 7.8, 1% Triton X100, 0.1% SDS, 250 mM NaCl, 5 mM EDTA, 100 mM NaF, 2 mM NaPPi, 2 mM Na3VO4, 1 mM PMSF) as described [35]. Cell lysates were separated on 12% SDS-PAGE and electrophoretically transferred to a polyvinylidene difluoride membrane (Bio-Rad, Hercules, CA, USA). Membranes were incubated overnight at 4° C with specific primary Abs (diluted 1/100, Santa Cruz Biotechnology, Dallas, TX, USA). Antibodies directed against tubulin, p-ERK, p-p70 S6 Kinase, LC3B, Beclin-1, p-Bcl-2, p-JNK, Caspase 3, Caspase 3-cleaved and Bax were purchased from Cell Signaling Technologies (Danvers, MA, USA). The presented protein blots were representative for three independent experiments. Immunocomplexes were visualized using the ECL detection system (Amersham Pharmacia Biotech, Little Chalfont, UK). Densitometric analysis of three independent experiments was performed for the quantification of the immunoblots, using the Molecular Analyst System program (Bio-Rad, Hercules, CA, USA).

### Immunoprecipitation

Total cell lysate was generated by lysis of cells with 1% NP40 (Sigma-Aldrich, St. Louis, MO, USA) protein lysis buffer (50 mM Tris-HCl, pH 8, 150 mM sodium chloride, 0.5% sodium deoxycholate and 0.1% sodium dodecyl sulfate) with the addition of protease inhibitor cocktail, phosphatase inhibitor cocktails I and II (Sigma, St. Louis, MO, USA). For each sample, total cell lysate containing 120 μg protein was incubated with 5 μl of anti-Beclin or anti-Bax antibody (Cell Signaling Technologies, Danvers, MA, USA) in rotation overnight at 4° C. Then 30 μl of Protein G Agarose beads were added to the mix of protein lysate and anti-Beclin or anti-Bax antibody, and incubation was performed overnight at 4° C. The IP complex was washed 6 times with NP40 lysis buffer. The beads were re-suspended with protein loading dye, the proteins were separated on SDS-PAGE and, then immunoblotted with antibody against p-Bcl2 or p-EGFR (Cell Signaling Technologies, Danvers, MA, USA).

### Autophagy assay

Autophagy was determined by fluorescence microscopic detection of the autophagosomes formation, by using the Monodansylcadaverine (MDC) labeling assay (Sigma-Aldrich, St. Louis, MO, USA). Cells were grown on chamber slides, washed with phosphate-buffered saline (PBS) and fixed in 10% formalin solution for 10 min. Autophagic vacuoles were labeled with MDC by incubating cells with 0.05 mmol/l MDC in PBS at 37° C for 10 min. Following incubation, the cells were washed three times with PBS and immediately analyzed under a fluorescence microscope (BX50, Olympus).

### Confocal microscopy

The autophagy marker LC3B was analyzed by confocal microscopy detection (LSM 510 Meta, Zeiss, Oberkochen, Germany) using anti-LC3B antibody and nuclear-ID red stain (Cell Signaling Technologies, Danvers, MA, USA). Cells on coverslips were fixed with a 4% paraformaldehyde solution, permeabilized with 0.25% Triton X-100 (Sigma-Aldrich, St. Louis, MO, USA) and incubated with primary and corresponding secondary antibodies (Alexa Fluor 488 and/or Alexa Fluor 647 conjugated) (Invitrogen, Carlsbad, CA, USA). Mounting medium contained DAPI (4’6-diamidino-2-phenylindole) to visualize the nucleus (Invitrogen, Carlsbad, CA, USA). Images of each experiment were acquired at the same exposure times within the same imaging session.

### ELISA

The concentration of EGF and VEGF released in the supernatants was determined using enzyme immunoassays (Wuhan Fine Biotech Co., Ltd., Wuhan City, China). The samples and the biotin conjugated detection antibody were subsequently added to the wells, pre-coated with anti-EGF or anti-VEGF antibody, and washed with wash buffer. Streptavidin was added and unbound conjugates were washed away with wash buffer. TMB substrates were used to visualize HRP enzymatic reaction. The O.D. absorbance at 450 nm was analyzed in a microplate reader (Infinite F50, Tecan Group Ltd., Mannedorf, Switzerland) and the concentration of EGF and VEGF were calculated, respectively.

### RNA isolation and real-time PCR

Total RNA was isolated using the Ribospin Kit (GeneAll Biotechnology Co., LTD, Seoul, Korea). Total RNA was reverse transcribed into cDNA using 1^st^ Strand cDNA Synthesis System (Origene, Rockville, MD, USA) and a real-time PCR (qPCR, Rotor-Gene 6000 Series 5 plex, Corbett Research, Mortlake, NSW, AU) was performed using Prime Time Mini qPCR Assay (IDT, Coralville, IA, USA). Expression of EGF was normalized using the GAPDH housekeeping gene and all reactions were performed in triplicate. The following primers were used: EGF, forward, 5′-CATCCTCTCCCTCTGAAATAC AC-3′; reverse, 5′-ACAGAATCTCAACACATGCTAG T-3′; probe, 5′-/56-FAM/AGCACAGTC/ZEN/ATCTTG ATCTGACACCATG/3IABkFQ/-3′. GAPDH, forward, 5′-TGTAGTTGAGGTCAATGAAGGG-3′; reverse, 5′-A CATCGCTCAGACACCATG-3′; probe, 5′-/56-FAM/AA GGTCGGA/ZEN/GTCAACGGATTTGGTC/3IABkFQ/ -3′ (IDT, Coralville, IA, USA). In order to perform gene quantification analysis, the Pfaffl method was used [36].

### Statistical analysis

Data presented as bar graphs represent the mean values of three independent data points. The error bars represent the standard deviation. Data are expressed as means ± SEM. Student *t*-test or ANOVA followed by the appropriate post-hoc test was used (Stat View 4.0 software, Abacus Concepts, Berkeley, CA, USA) as indicated in the respective figure legends. P values < 0.05 were considered statistically significant, with a confidence interval of 95%.

## SUPPLEMENTARY MATERIALS FIGURES AND TABLES



## Abbreviations

AMPK: AMP-activated protein kinase
Atg: Autophagy-related protein
Bax: Bcl-2 associated X protein
Bcl-2: B-cell lymphoma 2
CASPASE: cysteine-dependent aspartate-specific protease
cDNA: complementary DNA
DAPI: 4′6-diamidino-2-phenylindole
DMEM: Dulbecco's Modified Eagle Medium
ECL: Enhanced chemiluminescence
EDTA: Ethylenediaminetetraacetic acid
EGF: Epidermal growth factor
ERK: Extracellular signal-regulated kinase
FBS: Fetal bovine serum
FITC: Fluorescein isothiocyanate
GABARAP: GABA Type A Receptor-Associated Protein
GAPDH: Glyceraldehyde-3-Phosphate Dehydrogenase
HRP: horseradish peroxidase
JNK: c-Jun N-terminal kinase
MAPK: mitogen-activated protein kinases
MDC: Monodansylcadaverine
MK2: mitogen-activated protein kinase-activated protein kinase 2
MK3: mitogen-activated protein kinase-activated protein kinase 3
mTOR: Mammalian target of rapamycin
MTT: 3-(4,5-dimethylthiazol-2-yl)-2
Na3VO4: Sodium orthovanadate
NaCl: Sodium chloride
NaF: Sodium fluoride
NaPPi: sodium pyrophosphate
NP40: Nonidet P-40
O.D.: optical density
p70S6 kinase: Ribosomal Protein S6 Kinase B1
PBS: Phosphate-buffered saline
PMSF: Phenylmethanesulfonyl fluoride
qPCR: quantitative polymerase chain reaction
RNA: Ribonucleic acid
RPMI: Roswell Park Memorial Institute medium
SDS-PAGE: Sodium Dodecyl Sulphate - PolyAcrylamide Gel Electrophoresis
SEM: Standard error of the mean
TMB: 3,3′,5,5′-Tetramethylbenzidine substrate
Tris-HCl: Tris(hydroxymethyl)aminomethane hydrochloride
ULK: Unc-51 Like Autophagy Activating Kinase
VEGF: Vascular endothelial growth factor
VPS34: Vacuolar Protein Sorting 34.
