# Supplementary material for: Autophagy processes are dependent on EGF receptor signaling
Source: Oncotarget. 2018 Jul 13;9(54):30289–303. doi: 10.18632/oncotarget.25708 (PMC6084397; doi:10.18632/oncotarget.25708)
Supplement: Supplementary file 1 [file oncotarget-09-30289-s001.pdf]

# Autophagy processes are dependent on EGF receptor signaling

## SUPPLEMENTARY MATERIALS

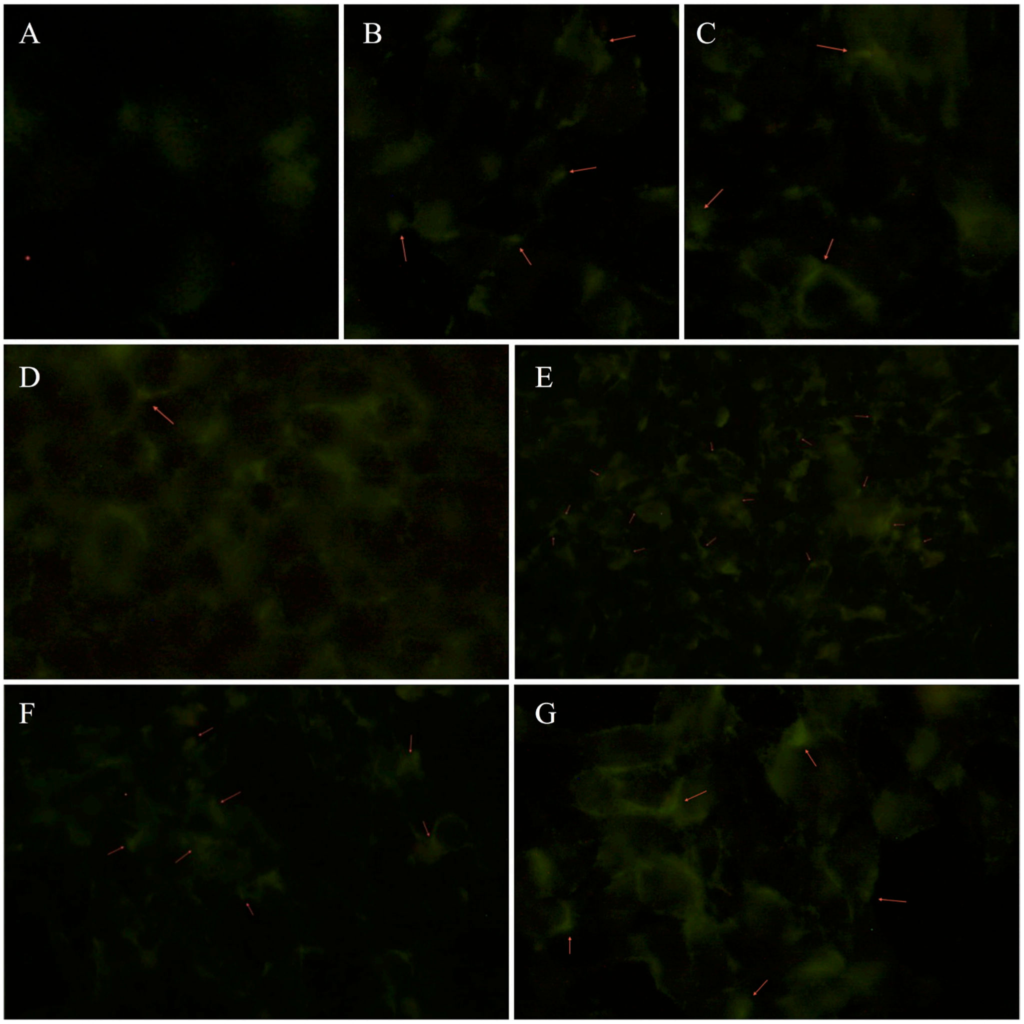


**Supplementary Figure 1: Autophagosomes formation analysis during starvation or Rapamycin treatment in 793 cell line.** Autophagy was determined by fluorescence microscopic detection of autophagosomes formation using the Monodansylcadaverine (0.05 mmol/l MDC) labeling assay. As well as for M14 cell line, these pictures evidence an increased presence of autophagosomes (red arrows) in 793 cells either undergoing starvation for 4 hrs (**B**), 12 hrs (**C**), 24 hrs (**D**) or treated with 10 (**E**) and 50 nm of Rapamycin (**F**) compared to control group (**A**). Moreover, cells treated with 100 nM Rapamycin (**G**) seem to be undergoing apoptotic processes.

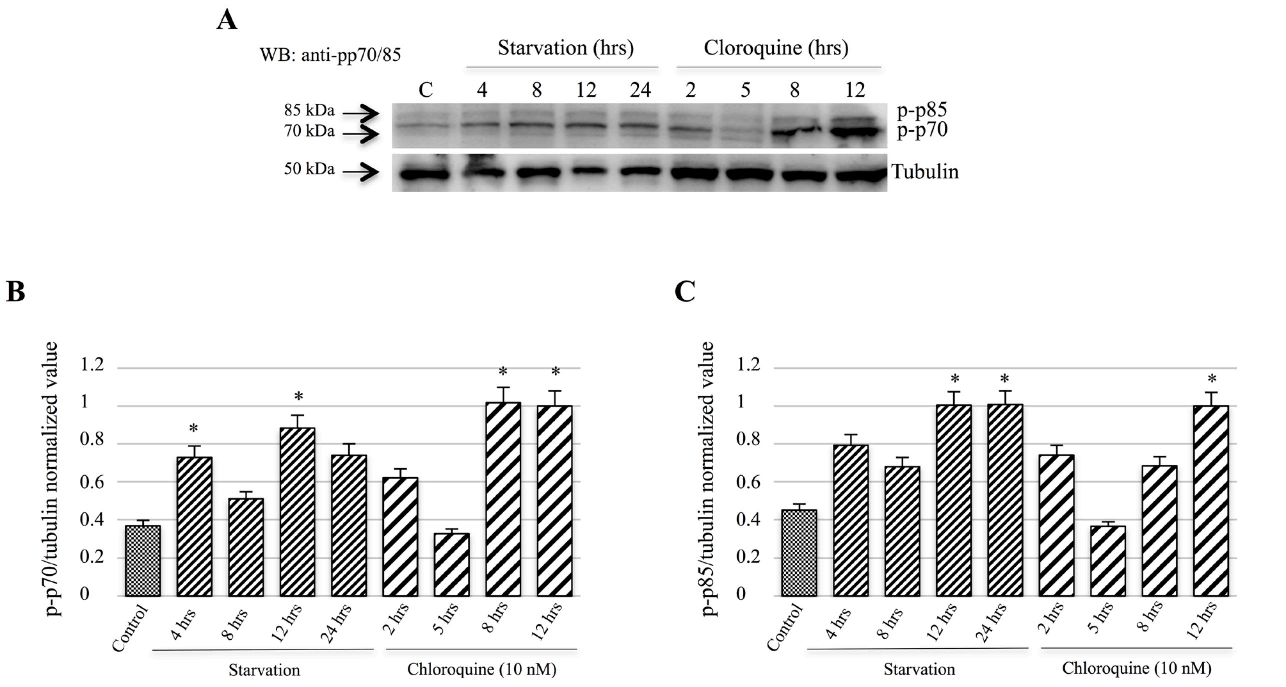


**Supplementary Figure 2: p-p70 and p-p85 S6 Kinase proteins expression were induced during starvation on 793 cells.** p-p70 and p-p85 S6 Kinase proteins expression were evaluated by Western blot analysis (**A**). As far as concern p-p70 protein expression, 793 cells starved for 4 and 12 hrs showed statistically significant differences compared to the control group (**B**). Furthermore, Western blot analysis showed a significant difference between controls and cells treated with Chloroquine for 8 and 12 hrs (B). As for as concern p-p85 protein expression, a significant increase among the groups starved for 12 and 24 hrs was observed as compared to the controls (**C**). Time of exposure of p-70/85 and tubulin were 13 and 4.3 s, respectively. $^{*}p < 0.05$ as compared to controls

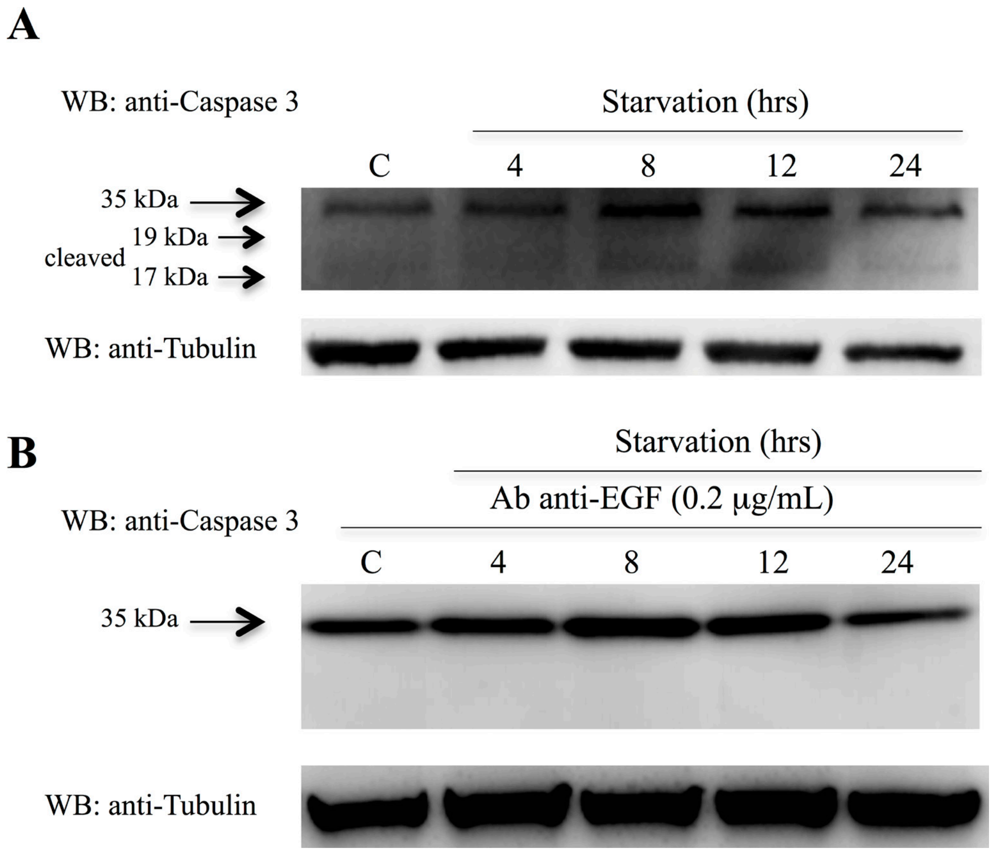


**Supplementary Figure 3: Evaluation of early and late apoptosis on M14 cells starved with or without anti-EGF antibody.** As previously observed on 793 cell line, the Caspase-3 cleaved expression was modulated on M14 cells starved for several different times, especially for 12 and 24 hrs (**A**), but not on cells treated with starvation and anti-EGF antibody (**B**). Time of exposure of Caspase-3 and tubulin were 4.3 s for both.
